# A Review of Indications and Technical Considerations of Endoscopic Balloon Dilation for Pediatric Subglottic Stenosis

**DOI:** 10.3390/jcm15082940

**Published:** 2026-04-13

**Authors:** Juma Obayashi, Manabu Komori, Yuri Nishiya, Nayu Yokoyama, Tomoko Kanno, Maho Wada, Kotaro Morita, Kosuke Kudo, Kunihide Tanaka, Shigeyuki Furuta

**Affiliations:** 1 Department of Pediatric Surgery, St. Marianna University School of Medicine, 2-16-1 Sugao, Miyamae, Kawasaki 216-8511, Kanagawa, Japan; yuri.nishiya@marianna-u.ac.jp (Y.N.); maho.wada@marianna-u.ac.jp (M.W.); kotaro.morita@marianna-u.ac.jp (K.M.); kosuke.kudo@marianna-u.ac.jp (K.K.); k3tanaka@marianna-u.ac.jp (K.T.); its0408@marianna-u.ac.jp (S.F.); 2 Department of Otolaryngology, St. Marianna University School of Medicine, 2-16-1 Sugao, Miyamae, Kawasaki 216-8511, Kanagawa, Japan; manabu.komori@marianna-u.ac.jp (M.K.); nayu.yokoyama@marianna-u.ac.jp (N.Y.); tomoko.kanno86@marianna-u.ac.jp (T.K.)

**Keywords:** subglottic stenosis, pediatrics, endoscopy, balloon dilation, technical procedure, balloon size, inflation pressure

## Abstract

Pediatric subglottic stenosis (SGS) remains a significant cause of upper airway obstruction in infants and children, most commonly resulting from prolonged endotracheal intubation. Although open airway reconstruction procedures such as laryngotracheal reconstruction (LTR) and partial cricotracheal resection (PCTR) provide definitive treatment for severe disease, their invasiveness and the burden of postoperative management have prompted increasing interest in less invasive approaches. Endoscopic balloon dilation has emerged as an effective therapeutic option, particularly for early-stage, short-segment, and soft acquired stenosis. This review summarizes the pathophysiology, clinical presentation, and severity assessment of pediatric SGS, and discusses the evolution of surgical management with particular emphasis on the role of balloon dilation. Technical aspects—including balloon sizing, adjunctive radial incision, dilation protocols, and local pharmacologic therapies—are outlined. Endoscopic balloon dilation is most effective in carefully selected patients, particularly those with early-stage and less severe stenosis. While it can significantly reduce the need for open airway reconstruction, recurrence remains a key limitation, necessitating careful patient selection and long-term follow-up. This article represents a narrative review of the current literature combined with the authors’ clinical experience.

## 1. Introduction

Pediatric subglottic stenosis (SGS) is defined as narrowing of the airway between the vocal folds and the inferior border of the cricoid cartilage [1]. In infants and young children, the subglottic region represents the narrowest portion of the airway and is encircled by the cricoid cartilage, the only complete cartilaginous ring in the respiratory tract. Consequently, even minimal mucosal edema or thickening may result in clinically significant airway obstruction. In recent years, endoscopic balloon dilation (EBD) has gained increasing attention as a minimally invasive alternative to open airway reconstruction. Although numerous studies have reported its effectiveness, the indications, technical approaches, and clinical outcomes remain variable across institutions. Therefore, a comprehensive synthesis of the current evidence is needed to clarify its role in the management of pediatric subglottic stenosis and to provide practical guidance for clinicians.

## 2. Literature Search Strategy

To summarize the current evidence regarding EBD for pediatric subglottic stenosis, a literature search was performed using PubMed and Google Scholar databases for articles published in English up to 2025. Search terms included combinations of the following keywords: “subglottic stenosis,” “pediatric,” “balloon dilation,” “endoscopic treatment,” and “airway stenosis.” Clinical studies, systematic reviews, and technical reports focusing on balloon dilation for pediatric airway stenosis were preferentially included. Reference lists of relevant articles were also screened to identify additional studies. This article represents a narrative review of the literature combined with the authors’ clinical experience.

## 3. Etiology and Epidemiology

Pediatric SGS is broadly classified as congenital or acquired based on its underlying pathogenesis. Congenital SGS accounts for approximately 10% of cases and results from failure of laryngeal recanalization during embryonic development (around the 10th week of gestation) [1]. It may present as either membranous or cartilaginous narrowing and is frequently associated with congenital conditions such as 22q11.2 deletion syndrome, Down syndrome, and CHARGE syndrome [2,3]. In contrast, approximately 90% of cases are acquired, most commonly as a consequence of prolonged endotracheal intubation. Sustained mechanical pressure from an inappropriately sized endotracheal tube can lead to mucosal edema, ulceration, and ischemic necrosis. During the healing process, excessive granulation tissue formation may occur, ultimately resulting in irreversible fibrotic scar tissue and progressive luminal narrowing [4]. Additional risk factors include gastroesophageal reflux disease, airway infection, and external trauma [5]. The incidence of acquired SGS increased markedly following the widespread adoption of prolonged neonatal intubation and mechanical ventilation in the 1960s [1,5]. However, improvements in ventilatory management and more careful selection of endotracheal tube size have led to a decline in incidence. Among neonates requiring prolonged intubation, the reported incidence is now approximately 1–2% [6,7]. In clinical practice, distinguishing congenital from acquired SGS can be challenging. Clear diagnostic criteria for cricoid hypoplasia remain lacking, and membranous stenosis may also be observed in patients presumed to have acquired disease. In cases of severe SGS, it is often difficult to determine whether stenosis is purely secondary to neonatal intubation or whether preexisting congenital narrowing contributed to intubation difficulty and subsequent worsening. This diagnostic overlap has important implications for treatment selection and prognosis.

## 4. Clinical Presentation

The clinical manifestations of SGS vary according to the degree of stenosis. The most common presenting symptom is stridor. Subglottic stenosis typically produces biphasic stridor, audible during both inspiration and expiration [4]. Increased airway resistance may also result in intercostal retractions, nasal flaring, and labored breathing [1]. Additional suggestive findings include a barking cough and recurrent croup-like episodes [8]. Mild stenosis may present as exertional dyspnea or recurrent respiratory infections mimicking pseudocroup. In contrast, severe stenosis may manifest as acute airway obstruction shortly after birth or as extubation failure in the intensive care setting, necessitating urgent airway intervention [1,8].

## 5. Severity Assessment

**Figure 1:**
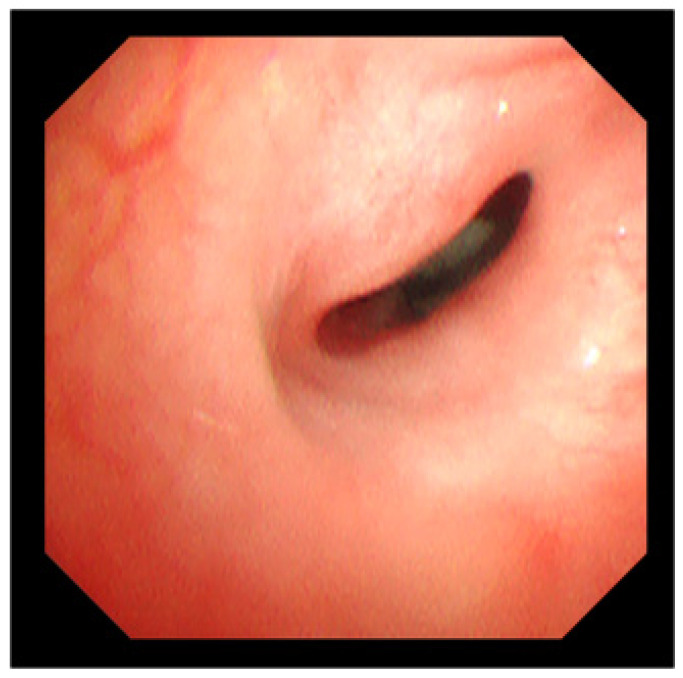
Preoperative flexible endoscopic view. A 2-year-old girl with subglottic stenosis classified as Cotton–Myer grade III.

SGS severity is most commonly evaluated using the Cotton–Myer classification based on ETT leak testing [9]. This system estimates the degree of luminal obstruction relative to the expected age-appropriate ETT diameter and plays a central role in guiding treatment decisions. Grade I: ≤50% stenosis, Grade II: 51–70% stenosis, Grade III: 71–99% stenosis, Grade IV: complete obstruction without detectable lumen (Figure 1). The subglottic region is physiologically the narrowest segment of the pediatric airway. In full-term neonates, a subglottic diameter of ≤4 mm is generally considered stenotic, whereas in premature neonates, a diameter of ≤3 mm is regarded as abnormal [1].

## 6. Evolution of Surgical Management for Pediatric Subglottic Stenosis

Surgical management of pediatric subglottic stenosis (SGS) has evolved over several decades, primarily with the goal of achieving decannulation and avoiding prolonged intubation or permanent tracheostomy. Prior to the widespread adoption of EBD, open airway reconstruction represented the principal definitive therapeutic approach [9]. To clarify the current role of balloon dilation, the established open surgical techniques—laryngotracheal reconstruction (LTR) and partial cricotracheal resection (PCTR)—are briefly reviewed below.

### 6.1. Laryngotracheal Reconstruction (LTR)

LTR involves incising the stenotic cricoid cartilage and expanding the airway lumen through placement of an autologous cartilage graft, most commonly harvested from the rib [8]. This procedure is typically indicated for moderate stenosis, corresponding to Cotton–Myer grade II or III lesions [10]. In cases of more advanced stenosis (late grade III to IV), particularly when extensive mucosal loss is present, the risk of granulation tissue formation and restenosis at the graft site increases, potentially compromising surgical outcomes [11]. LTR is generally categorized into two approaches based on postoperative airway management:

- Single-stage LTR: The tracheostomy is closed at the time of reconstruction (or no tracheostomy is present initially). An endotracheal tube is maintained postoperatively as an internal stent for approximately 7–10 days [12].
- Double-stage LTR: The tracheostomy is maintained during reconstruction, and a stent such as a T-tube or LT-Mold is placed for at least six weeks before decannulation is attempted [13].

### 6.2. Partial Cricotracheal Resection (PCTR)

PCTR is a more definitive surgical procedure in which the stenotic segment—including the anterior cricoid arch and involved tracheal rings—is circumferentially resected, followed by primary end-to-end anastomosis of healthy airway segments [11]. It is typically indicated for severe stenosis (Cotton–Myer grade III–IV) or as a salvage procedure following failed LTR [10,14]. Because fibrotic scar tissue is completely excised, reported decannulation rates range from 90% to 96%, often exceeding those of LTR [13]. However, the procedure is technically demanding, particularly in pediatric patients, requiring extensive mobilization of the trachea to minimize tension at the anastomotic site [10,13].

### 6.3. Limitations of Open Surgery and the Shift Toward Endoscopic Approaches

Although LTR and PCTR provide reliable solutions for severe SGS, both procedures carry significant risks. In PCTR, anastomotic dehiscence may result in life-threatening airway compromise or mediastinitis [10,11]. Additional potential complications include recurrent laryngeal nerve injury, graft displacement in LTR, granulation tissue formation, infection, and tissue necrosis [13]. Revision LTR may be technically challenging due to tissue fragility, and reoperation after failed LTR often necessitates conversion to PCTR. Furthermore, complications following PCTR can be particularly difficult to manage. Postoperative care following open reconstruction is also intensive. In single-stage LTR, prolonged postoperative intubation and deep sedation in the pediatric intensive care unit are required to maintain airway stability. This approach carries risks such as accidental extubation, ventilator-associated pneumonia, and withdrawal symptoms, and is associated with extended hospitalization [15]. These inherent risks and the substantial perioperative burden of open reconstruction have contributed to increasing interest in less invasive alternatives. Consequently, EBD has gained prominence as a potential strategy to reduce surgical morbidity while preserving airway patency in selected patients.

## 7. Endoscopic Balloon Dilation

In this section, the indications, limitations, and practical considerations of EBD are discussed, incorporating both published evidence and our institutional experience.

### 7.1. Indications and Limitations of Balloon Dilation

EBD can be broadly categorized into three clinical roles: primary treatment, adjunctive therapy, and an alternative to open surgical intervention, depending on disease severity and characteristics. As a primary treatment, EBD is most effective for acquired, low-grade (Cotton–Myer grade I–II) subglottic stenosis, particularly in early or immature lesions following recent intubation. As an adjunctive therapy, EBD is commonly used following open airway reconstruction to manage restenosis or granulation tissue formation. In contrast, open surgical procedures such as laryngotracheal reconstruction or cricotracheal resection are generally preferred in patients with high-grade stenosis, mature fibrotic lesions, or anatomically complex disease.

EBD for pediatric SGS can achieve favorable outcomes when patients are appropriately selected. The success of this technique depends largely on the histologic characteristics and anatomical configuration of the stenotic segment, making accurate preoperative assessment essential. The literature consistently emphasizes that balloon dilation is most effective in cases involving immature, soft stenotic tissue. Specifically, Cotton–Myer grade I and II stenoses are considered the primary indications [1,4,14]. Although the procedure has been attempted in grade III lesions, success rates decrease significantly with increasing severity of obstruction [4]. From a pathophysiological perspective, the most favorable indication is early acquired SGS diagnosed within 30 days after extubation, before scar maturation has occurred [4]. Thin, membranous (“weblike”) stenoses that are amenable to radial expansion also respond well to balloon dilation [1,10]. Conversely, several conditions are associated with limited efficacy. Congenital SGS, particularly when characterized by thickened or rigid cricoid cartilage, often responds poorly to intraluminal expansion alone [1,4]. Similarly, long-standing acquired stenosis with mature, dense fibrosis is less likely to expand adequately [1,4]. Anatomical abnormalities such as elliptical cricoid deformity, cartilage deficiency [1], or long-segment stenosis extending into the trachea [1,4] further reduce the likelihood of success. In such cases, open airway reconstruction, including LTR or PCTR, is generally recommended [4]. In summary, balloon dilation offers the greatest benefit in relatively mild stenosis (up to grade II), particularly when intervention is performed during the early phase of acquired disease before scar maturation. Because prolonged postoperative stenting is typically unnecessary, the procedure may be performed even in younger patients and can be repeated if restenosis occurs. Based on both published data and our institutional experience, balloon dilation may be considered as an initial minimally invasive option in selected tracheostomized patients, provided that anatomical suitability is carefully confirmed.

### 7.2. Technical Procedure

Although EBD is minimally invasive, it requires meticulous technique and adherence to standardized protocols. The practical aspects of the procedure are outlined below.

#### 7.2.1. Balloon Size Selection

Appropriate balloon size selection is critical to maximize dilation efficacy while minimizing the risk of serious complications such as airway rupture. In general, balloon diameter is determined based on the estimated normal airway size for the patient’s age and body size. The selection of balloon diameter is standardized to approximately 2 mm larger than the outer diameter of an age-appropriate endotracheal tube (ETT) [1,4,16,17]. In another report, in a 4-year-old child for whom a 5.0 mm internal diameter ETT (outer diameter approximately 7 mm) is considered appropriate, a 7–8 mm balloon has been reported to be suitable [1]. Some authors recommend a minimum balloon diameter of 6 mm [7]. Balloon dilation catheters are typically thin and highly flexible, allowing safe passage through even severely narrowed segments and precise positioning at the target site [1].

#### 7.2.2. Adjunctive Radial Incision

Balloon dilation alone may be insufficient in cases of fibrotic stenosis due to the elastic recoil of scar tissue, which can predispose to early restenosis. For this reason, adjunctive radial incision of the stenotic segment is frequently performed prior to balloon inflation to enhance expansion. For thick or mature scars, many clinicians advocate for adjunctive radial incisions using a cold knife or laser prior to dilation to mechanically interrupt scar continuity [17,18]. By disrupting the continuity of dense scar tissue, radial incisions facilitate more effective luminal enlargement [1]. Incisions may be created using a sickle knife, microlaryngeal scissors, or laser devices such as CO_2_ or KTP lasers [1,2]. In cases of recurrent stenosis, the combination of CO_2_ laser radial incision with balloon dilation has been reported to significantly prolong the treatment-free interval compared with balloon dilation alone [9].

#### 7.2.3. Dilation Protocol (Inflation Pressure, Duration, and Frequency)

**Figure 2:**
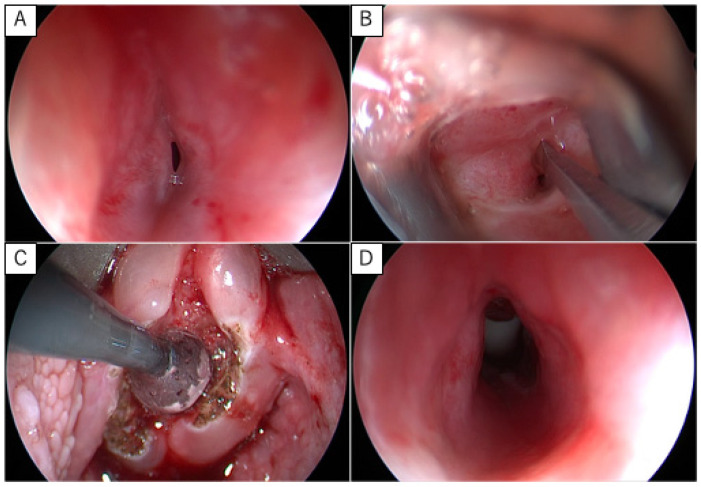
Endoscopic balloon dilation procedure. (**A**) Direct laryngoscopy under rigid endoscopic visualization demonstrating grade III subglottic stenosis, consistent with findings on flexible endoscopy. (**B**) Radial incisions were made in the stenotic segment using a vocal fold retractor to expose the subglottic region. (**C**) Sequential balloon dilation was performed: a 10-mm balloon was inflated to 10 atm for 30 s three times and for 60 s once. Concomitant CO_2_ laser ablation was performed for associated laryngomalacia. (**D**) Immediate post-dilation view showing improved airway patency with controlled bleeding.

Although protocols vary among institutions, certain standardized principles are widely adopted. Inflation pressures vary between institutions, with reports ranging from 8 to 12 atmospheres up to 16 atmospheres [19,20], or as recommended by the manufacturer [4]. The duration of each dilation cycle is closely related to intraoperative airway management under general anesthesia. Many protocols recommend maintaining dilation for up to 2 min, provided that oxygen desaturation does not occur [1,4]. Alternatively, some protocols limit each dilation to 30 s or terminate inflation when oxygen saturation falls below 92% [7,21,22]. Dilation is typically repeated two to three times within a single operative session (cyclic dilation) [4,7,23]. Depending on postoperative response, repeated sessions may be scheduled at intervals of 1 to 3 weeks until clinical stability is achieved [1,18]. Airway management during the procedure requires close coordination with the anesthesia team, particularly in non-tracheostomized patients with mild SGS (grade I–II), where dilation and airway maintenance must be alternated. Although ventilation via rigid bronchoscopy is ideal, the short distance between the vocal cords and the stenotic segment often makes simultaneous stabilization of both the rigid scope and the balloon technically challenging. Therefore, intermittent mask ventilation or repeated endotracheal intubation may be necessary. In contrast, in tracheostomized patients, airway control is secured, allowing safer and more controlled dilation. In our institution, prioritizing patient safety—particularly in cases with unstable respiratory status—we generally perform balloon dilation after tracheostomy when indicated. Balloon placement is achieved either under microscopic visualization using a laryngoscope or under endoscopic guidance (Figure 2).

#### 7.2.4. Local Pharmacologic Adjuncts for Restenosis Prevention

Restenosis remains a major challenge following balloon dilation. Therefore, various adjunctive pharmacologic therapies are commonly administered intraoperatively. Topical application of mitomycin C (MMC) is used to inhibit fibroblast proliferation and reduce scar formation. Reported protocols include application of 0.5 mg/mL MMC for 2 min [24] or 5 min [25]. Submucosal steroid injection is also frequently employed to reduce inflammation and scar formation. Methylprednisolone acetate (10 mg/mL, 1–2 mL) or triamcinolone (40 mg/mL, 2 mL) may be injected circumferentially into the stenotic segment either before or after dilation or incision [10,26]. Additionally, topical application of steroid–antibiotic ointments has been described. One approach involves applying the medication locally for 1–2 min immediately after dilation [7]. Another technique includes temporary reintubation for approximately 48 h using a smaller ETT coated with betamethasone (0.5 mg) and gentamicin (1 mg) ointment, functioning as a short-term internal splint [23].

### 7.3. Clinical Outcomes and Prognosis

#### 7.3.1. Treatment Outcomes (Decannulation and Tracheostomy Avoidance)

The reported success rates for EBD in acquired pediatric SGS generally range from 65% to 86% [17,18,19,20,21,22,23,27,28] (Table 1). When applied to appropriately selected patients, balloon dilation has demonstrated favorable clinical outcomes and is estimated to reduce the need for open airway reconstruction by approximately 80% [4]. In particular, acquired SGS diagnosed within 30 days after extubation—before scar maturation—has been associated with reported success rates of up to 100% [4]. In cases of thin, acute membranous stenosis, success rates can reach 100%, often precluding the need for a tracheostomy [18,28]. In contrast, in chronic acquired SGS diagnosed more than 30 days after extubation, where fibrotic maturation has occurred, success rates decrease to approximately 39% [4]. A meta-analysis with a mean follow-up period of 4.6 months reported an overall success rate of 65.3% (range, 50–100%) [20]. In studies focusing specifically on acquired SGS, success rates of 82.3–86% have been described [19,22]. In addition, adjunctive strategies such as temporary internal splinting using a steroid- and antibiotic-coated endotracheal tube have demonstrated tracheostomy avoidance in approximately 85% of cases (50 of 59 patients) [23]. Successful restoration of airway patency has been associated with improvements in physical, emotional, and social domains of quality of life over long-term follow-up [7].

#### 7.3.2. Complications and Risk Management

Although balloon dilation is generally regarded as a safe and minimally invasive procedure, it is not without risk. The most common postoperative complications include transient mucosal edema and restenosis. Postoperative edema may temporarily exacerbate airway narrowing and requires careful monitoring [29]. Restenosis remains the principal limitation of this technique, with long-term recurrence rates reported between 51.6% and 59.2% in some series [7,9]. Even when initial improvement is achieved, structured follow-up is essential to detect recurrence early and to allow timely repeat dilation when appropriate. EBD is characterized by a high need for repeated procedures, with an average of 1.6 to 3 dilations required per patient [21,22,27]. Recurrence rates are significantly higher for chronic, thick scars and high-grade stenoses [19]. Factors consistently associated with treatment failure include patient weight < 5 kg, the presence of comorbidities (especially GERD), cartilaginous framework abnormalities, and multiple concomitant airway lesions [1,20,21,23]. Rare but potentially serious complications have also been reported. Excessive dilation may result in airway rupture, mucosal injury, or laceration [7,30], which can be complicated by pneumothorax or pneumomediastinum [4,13,30]. Intraoperative complications related to inadequate airway control, including laryngospasm and oxygen desaturation, may occur in the absence of meticulous anesthetic management [23]. Very rarely, balloon entrapment within the airway has been described [30], underscoring the importance of procedural vigilance and readiness to respond to unexpected events. Despite its minimally invasive nature, balloon dilation has several important limitations. Recurrence remains a significant concern, and multiple procedures are often required to achieve sustained airway patency. Reported success rates vary widely across studies, particularly depending on stenosis severity and tissue characteristics. Chronic, mature, or high-grade stenosis is associated with lower success rates and a higher likelihood of treatment failure. Furthermore, a subset of patients ultimately requires additional surgical intervention, including open airway reconstruction such as laryngotracheal reconstruction or cricotracheal resection.

## 8. Conclusions

EBD represents a valuable minimally invasive option in the management of pediatric subglottic stenosis, particularly in carefully selected patients with early-stage and low-grade disease. Appropriate patient selection and timely intervention are critical to achieving favorable outcomes. While balloon dilation can reduce the need for open airway reconstruction, its effectiveness is limited in high-grade or mature stenosis, and repeated procedures are often required. Future research should focus on standardizing treatment protocols, identifying predictors of success, and clarifying long-term outcomes to optimize patient management and improve clinical decision-making.

## Figures and Tables

**Table 1:** Summary of Clinical Outcomes and Procedural Characteristics of Endoscopic Balloon Dilation for Pediatric Subglottic Stenosis.

Author	Year	Study Design	Patient Number	SGS Grade	Number of Dilations (Mean)	Success Rate
Hu [17]	2024	Retrospective	33	I–III	1.88	72.7%
Sahin Onder [18]	2020	Retrospective	41	I–III	1.8 (Overall)	100% (Thin)/40% (Thick)
Kurdi [19]	2019	Retrospective	10	II–III	3.0	86%
Lang [20]	2014	Meta-analysis	150	I–III	1.9	65.3%
Wentzel [21]	2014	Meta-analysis	202	I–III	2.26	64%
Lira [23]	2025	Retrospective	59	I–III	Median 1 (range 1–6)	84.7% (avoided tracheostomy)
Powell [27]	2020	Registry (AIR)	59	I–III	2.25	65% (Primary)
Avelino [28]	2014	Retrospective	9	I–III	2.5	100% (Acute)

SGS: subglottic stenosis; AIR: Airway Intervention Registry. SGS grade is based on the Cotton–Myer classification. “Number of dilations” represents the mean number of procedures per patient unless otherwise specified. Success rate definitions vary among studies and generally include avoidance of tracheostomy, decannulation, or improvement in airway patency. “Thin” and “thick” refer to the histologic characteristics of the stenotic lesion. “Primary” indicates balloon dilation used as an initial treatment strategy.

## Data Availability

The original contributions presented in this study are included in the article. Further inquiries can be directed to the corresponding author.

## Abbreviations

The following abbreviations are used in this manuscript:

EBD	Endoscopic balloon dilation
SGS	Subglottic stenosis
LTR	Laryngotracheal reconstruction
PCTR	Partial cricotracheal resection
ETT	Endotracheal tube
MMC	Mitomycin C
